# Establishment and evaluation of a general dissociation technique for antibodies in circulating immune complexes

**DOI:** 10.1007/s10238-018-0523-4

**Published:** 2018-08-17

**Authors:** Tong Wang, Meng Zhang, Huajun Zhou, Dawei Cui, Xujian Xu, Changgui Sun, Yuzhu Dai, Jun Cheng

**Affiliations:** 1 Faculty of Graduate Studies, Bengbu Medical College, Bengbu, China; 2 Department of Clinical Laboratory Science, The 117th Hospital of PLA, 14 Lingyin Road, Westlake District, Hangzhou, 310013 China; 3 State Key Laboratory for Diagnosis and Treatment of Infectious Diseases, First Affiliated Hospital, Zhejiang University Medical College, Hangzhou, China; 4 Department of Biotechnology, The University of Tokyo, Tokyo, Japan

**Keywords:** Antibody dissociation, Circulating immune complex, Dissociation technique, Immune complex

## Abstract

This study aimed to establish a general and efficient dissociation technique for detecting antibodies in circulating immune complexes (CICs) in serum and to evaluate its clinical application. CICs were efficiently separated from specimens using polyethylene glycol double-precipitation. The best conditions for anti-HBs dissociation from HBsAg-ICs were a pH of 1.80, incubation at 15 °C for 5–10 min, and detection within 10 min after neutralization. The mean dissociation rate, reproducibility, mean dissociation recovery rate and specificity of the new technique were 64.3%, < 5.97, 95.4 and 100%, respectively. They had a favourable linear relationship (*r* = 0.9932), and the stability of the reagents exceeded 24 months, except the CIC antibody dissociation reagent (> 12 months). Conditions for the dissociation of other CICs tested were similar, but there were differences in the rate of antibody dissociation. Different HBV-M patterns had significantly different levels and rates of antibody dissociation from HBsAg-IC (*P* < 0.05), and the detection rates of the corresponding antibodies in HCV, core-anti-HCV core antibody (HCV-ICs), HIV P24-anti-HIV P24 antibody (HIV-ICs), insulin-anti-insulin antibody (INS-ICs) and thyroid globulin-anti-thyroid globulin antibody CICs (TG-ICs) were 34.8, 66.7, 20 and 14.3%, respectively. These data suggest that our CIC antibody dissociation technique is a good general pretreatment technique for the detection of antibodies after the precipitation, separation and dissociation of multiple CICs.

## Introduction

The invasion of pathogens, such as bacteria and viruses, into the body or alterations and the exposure of tissue components in the body may induce immune system responses, causing the production of specific immune effector cells and antibodies. These antibodies may specifically bind to antigens to form immune complexes (ICs), which are then cleared by the defence system to protect tissues against immune-induced damage [1–3]. However, under pathological conditions, the ICs formed in the body are not quickly removed, which may cause a series of injuries and result in clinical symptoms, known as immune complex disease [4–6]. Thus, the accurate detection of circulating ICs (CICs) in tissues and body fluid aids in diagnosis, monitoring disease conditions, determining therapeutic efficacy, assessing prognosis and investigating the pathogenesis of certain diseases, giving it clinicopathological and epidemiological significance [7–11].

The currently available methods for detecting CICs include the following: (1) the detection of total CICs via antigen-nonspecific methods (such as physical techniques, complement techniques, antiglobulin techniques, and cellular techniques) [12] and antigen-specific methods (such as in two-component-determined CICs) [13]; (2) antigen-specific methods for detecting antigens in CICs (such as the HCl dissociation technique [14], the surfactant dissociation technique [15], the trypsin digestion technique [16], the immune complex transfer technique [17], immune complexome analysis [18] and the CIC antigen dissociation technique [19]); and (3) antibody-specific methods for detecting antibodies in CICs (such as the dissociation enzyme-linked immunosorbent assay (ELISA) for anti-Leishmania IgG in ICs [20]). The above methods, except for the CIC antigen dissociation technique [19], have poor specificity [12, 21] and poor sensitivity; therefore, these approaches may not meet the requirements for clinical diagnosis and scientific studies [12, 13, 15, 21] and may have limited applications [13–18, 20]. In particular, little is known about antibody-specific methods for detecting antibodies in CICs. Based on our previous findings [19] and previously reported studies [20], we developed a general and efficient dissociation technique to detect antibodies in CICs (also known as the antibody-specific method for detecting CICs). This technique can be used for dissociation of specific antibodies from CICs in serum samples. Specific antibodies are detected by corresponding methods or against different antigens in CICs. For example, chemiluminescence can be used to detect anti-hepatitis B surface antigen antibody (anti-HBs) dissociated from hepatitis B surface antigen (HBsAg)-anti-HBs ICs (HBsAg-ICs), anti-thyroglobulin (TG) antibody dissociated from TG-anti-TG antibody ICs (TG-ICs), and anti-insulin antibody dissociated from insulin-anti-insulin antibody ICs (INS-ICs). ELISA can be used to detect anti-hepatitis C virus (HCV) core antibody dissociated from HCV core-anti-HCV core antibody ICs (HCV-ICs) and anti-human immunodeficiency virus (HIV) P24 antibody dissociated from HIV P24-anti-HIV P24 antibody ICs (HIV-ICs). In this study, our novel technique for dissociating antibodies from CICs was assessed by detecting HBsAg-ICs as an example case, and relevant methodological parameters were preliminarily evaluated and applied.

## Materials and methods

### Sample collection

A total of 153 samples negative for HBsAg, anti-HBs, HBeAg, anti-HBe and anti-HBc (HBV-M-5, healthy volunteers as a negative control group) were collected from healthy volunteers undergoing routine physical examinations. For the disease group, a total of 850 serum samples were collected from hepatitis B virus (HBV)-infected patients, HCV-infected patients, HIV-infected patients, diabetes mellitus patients, patients with hyperthyroidism, and patients with other diseases through the Specimen Bank of our hospital and the First Hospital of Zhejiang University. The Specimen Bank contains materials related to infectious diseases and other common abnormal results. The “other disease” samples (*n* = 155) served as an interference control group. The basic information on the research subjects is shown in Table 1.

**Table 1:** The details of 1158 subjects

Types of subjects	Number of cases (*n*)	Description
Healthy population (negative control) HBV-M1	153	Negative for HBsAg, anti-HBs, HBeAg, anti-HBe, anti-HBc
HBV infection		
HBV-M2	108	Positive for HBsAg, HBeAg, anti-HBc
HBV-M3	165	Positive for HBsAg, anti-HBe, anti-HBc
HBV-M4	64	Positive for HBsAg, anti-HBc
HBV-M5	118	Positive for anti-HBs, anti-HBe, anti-HBc
HCV infection	126	Positive for anti-HCV
HIV infection	32	Positive for anti-HIV
Diabetics	103	Insulin treatment
Hyperthyroid	134	Positive for anti-TG
Patients with other diseases (interference control)	21	Hypertriglyceridaemia (9.51–23.2 mmol/L)
	13	Haemolysis (free haemoglobin: 3.2–6.4 g/L)
	33	Jaundice (total bilirubin: 224.5–486.5 µmol/L)
	10	High IgG immunoglobulin hyperlipidaemia (51.3–59.6 g/L)
	27	Strong positivity for anti-HCV antibody (S/CO = 9.7–17.3)
	9	Strong positivity for anti-HAV-IgM antibody (S/CO = 11.5–20.3)
	42	Strong positivity for rheumatoid factor antibody or antinuclear antibody and CIC (S/CO = 7.6–12.9)

### Main reagents and instruments

An Architect i2000 chemiluminescence immune analyser, the corresponding HBV-M reagents (HBsAg Quantitative II, anti-HBs, HBeAg, anti-HBe, and anti-HBc), HIV1/2 antigen/antibody, an insulin detection kit, and an anti-TG antibody kit were purchased from Abbott Diagnostics (USA); a DX800 chemiluminescence immune analyser and the corresponding TG detection kit were obtained from Beckman (USA). Other instruments used were a 3K18 low-temperature centrifuge (Sigma Co., LLC, Germany), an SHA-EA low-temperature water bath shaker (Jingda, China), an Osmomat 030 cryoscopic osmometer (Gonotec, Germany), and a PP-50-P11 professional pH metre (Sartorius, Germany).

The following assay kits were utilized: ELISA anti-HBs assay kits (InTec, China), an ELISA HCV core antigen assay kit (Kangrun, China), an HCV Version 3.0 ELISA Test System for detecting anti-HCV (Ortho, USA), an ELISA HIV P24 antigen assay kit (XpressBio, USA), Recombinant and synthetic peptide enzyme immunoassay (EIA) for detecting Anti-HIV-1/2 (Bio-Rad, USA), anti-insulin antibody (IAA) ELISA for detecting anti-insulin (DRG, USA), an ELISA anti-Clq capture assay kit for detecting human CIC (Euroimmun, Germany).

The antigens and antibodies used in the present study were as follows: purified HBV surface antigen from human plasma (3.0 mg/ml, Genia Life Sciences, Inc., China), goat anti-HBsAg polyclonal antibody (3.0 mg/ml, Acon Biotech Co. Ltd., China), recombinant HCV core (1 mg/ml, Ness-Ziona, Israel), goat anti-HCV core polyclonal antibody (5 mg/ml), recombinant HIV 1 P24 (1 mg/ml), goat anti-HIV 1 P24 polyclonal antibody (4 mg/ml), recombinant insulin (5 mg/ml), goat anti-insulin polyclonal antibody (0.2 mg/ml), goat anti-TG polyclonal antibody (1.5 mg/ml) (Cambridge, UK), purified human TG (1 mg, Maryland Heights, USA). The 0.25% trypsin -EDTA (1X) (Gibco, Canada), polyethylene glycol 6000 (Sigma-Aldrich, USA), and other chemical reagents purchased from Shanghai Chemical Reagents Co., Ltd.

### Preparation of HBsAg-IC and other ICs

HBsAg-IC solutions were prepared at appropriate concentrations according to the detection range of the HBsAg detection kit using an Architect i2000 chemiluminescence immune analyser. Goat anti-HBsAg polyclonal antibody was diluted to 500 mIU/ml by isotonic 0.1 M phosphate-buffered saline (PBS, pH = 7.4) containing 10% calf serum. Purified human HBsAg was then added until the free antibody was < 15 mIU/ml and free HBsAg was < 1.5 IU/ml. The solution was incubated at 37 °C for 2 h and then at 4 °C overnight. Stable HBsAg-ICs formed, and the final concentration of anti-HBsAg antibody (anti-HBs) was 473.6 mIU/ml. After the addition of 0.02% ProClin 300 biological preservative, the solution was stored at 4 °C as positive control I. In addition, HBV-M-negative human serum was used to dilute goat anti-HBsAg polyclonal antibody to prepare HBsAg-ICs at an equal concentration via the same procedures, and the HBsAg-IC solution was then stored at 4 °C as positive control II. The HCV-IC, HIV-IC, INS-IC and TG-IC solutions were prepared as described above.

### Preparation of CIC dissociation reagents

CIC separating agent A, CIC separating agent B, and CIC dissolving agent were prepared as previously reported with slight modifications [19]. Briefly, to prepare the CIC separating agent, 0.15 M borate buffer containing 0.132 M NaCl and 0.02% ProClin 300 biological preservative was mixed with 8% and 7% polyethylene glycol (PEG) 6000 to yield CIC separating agent A and CIC separating agent B, respectively. The parameters for the quality control were a pH of 8.40–8.80 with the osmolarity at 500–530 osm. For the CIC dissolving agent, a mixture containing 0.154 M NaCl, 0.3% Triton X-100 and 0.02% ProClin 300 was mixed with 1N NaOH until the pH was 7.4. The parameters for the quality control were a pH of 7.30–7.50 with the osmolarity at 280–300 osm.

Four kinds of CIC antibody dissociation agents were prepared with 0.02% ProClin 300 biological preservative and 0.06 M glycine-HCl buffer at different pH values (pH: 1.0, 1.4, 1.8, and 2.2), regulating osmotic pressure through NaCl to iso-osmia (280–300 osm). The four kinds of CIC antibody dissociation neutralizing agents were prepared with different concentrations of Tris solution and 0.02% ProClin 300. After neutralization with the corresponding CIC antibody dissociation agents (pH: 1.0, 1.4, 1.8, and 2.2), the final pH values were 7.25–7.45, and the osmolarity values were 280–300 osm.

### Double-precipitation separation and antibody dissociation from CICs

Double-precipitation separation: One hundred fifty microlitres of CIC separating agent A was mixed with 150 μl serum in a 1-ml tube, followed by incubation at 37 °C for 30 min and then at 4 °C for at least 6 h. The mixture was then centrifuged at 29,000 g for 5 min or 4000 g for 20 min, and the supernatant was removed. One hundred fifty microlitres of 0.9% NaCl was added to the above CIC sediment, followed by vortexing until the granules were resolved. Then, 150 μl of CIC separating agent B was added, followed by incubation for at least 6 h at 4 °C. The mixture was centrifuged at 29,000 g for 5 min or 4000 g for 20 min. The supernatant was removed, and CIC sediment was reserved for use.

Antibody dissociation from CICs: The same two parts of HBsAg-CIC precipitation from double-precipitation separation were each dissolved by 10 μL solvent into two tubes, one of which was as a blank control for antibody determination. Seventy microlitres of a CIC antibody dissociation agent at one of the four pH values and 70 µl of a corresponding concentration of neutralizing agent were added to the blank control tube. After mixing, the antibody was detected, and the result was defined as the blank value. The other tube was the dissociated sample, which was used to detect the antibody of the dissociation of CIC. Seventy microlitres of a CIC antibody dissociation agent at one of the four pH values were added to the tube and were used to determine the dissociation under different temperatures, times and oscillation frequencies. When the dissociation finished, we added 70 µl of a corresponding concentration of neutralizing agent. After mixing, the antibody was measured after different incubation times. The result was recorded as the measured value. Each experiment was repeated three times, and the average result was calculated.

To ensure the process of antibody dissociation from CIC and measurement was stable and comparable, CIC antibody dissociation agent and solution after neutralizing were maintained in an isotonic state (regulated by NaCl). When the dissociation of the antibody from CIC was detected, the same volume and corresponding concentration of Tris were added as the “CIC antibody neutralization agent”, which returned the pH to 7.3–7.5 and the osmotic pressure of the reaction system to the isotonic state.

Interpretation of results: the criteria for determining antibody results in CIC are shown in Table 2.

**Table 2:** The criteria for classification of antibody after CICs dissociation

Measurement value	Blank control value	Explanation
> Cutoff^a^	< Cutoff	There were CICs in the sample, and their concentration was determined on the basis of the measurement value
< Cutoff	< Cutoff	There were no CICs in the sample
< Cutoff	> Cutoff	This pattern did not exist or suggest random error
> Cutoff	> Cutoff	If the measurement value was higher than the blank control value, there were CICs in the sample, and their concentration was determined on the basis of the difference. If the measurement value was lower than the blank control value, there were no CICs in the sample

^a^The cutoff value was obtained from the manufacturer’s instructions of each specific kit

### Comparison of the PEG double-precipitation separation method with the traditional PEG precipitation separation method

Four free HBsAg-positive samples (samples 1–4: 2.68–182.92 IU/ml), 3 free anti-HBs-positive samples (samples 5–7: 23.89–897.32 mIU/ml, of which sample 6 and 7 were from acute HBV-infected patients in the recovery phase), 1 sample (sample 8) negative for both free HBsAg and free anti-HBs, and the prepared HBsAg-IC positive control I and positive control II were independently deposited and separated using the PEG double-precipitation separation method [19] and the traditional PEG precipitation separation method [22]. The CIC antibody dissociation technique developed by our group was then used to dissociate anti-HBs from HBsAg-ICs and the corresponding blank control. Finally, an Architect anti-HBs quantitative kit was used to detect anti-HBs, and the efficacy of precipitation and separation was compared between the two techniques (each sample was detected ten times, and the average result was calculated).

### Optimization of conditions for anti-HBs dissociation from HBsAg-ICs

The dissociation of anti-HBs from HBsAg-ICs was performed according to a previous method [19], with slight modifications. Briefly, the HBsAg-IC positive control II was precipitated by the double-precipitation separation method under conditions including 0.06 M glycine-HCl buffer at different pH values (1.0, 1.4, 1.8 and 2.2), temperatures (4 °C, 15 °C, 25 °C and 37 °C), dissociation times (5 min, 8 min, 10 min and 20 min), shaking frequencies (0,20,60, and 100 cycles /min) and residence times after dissociation (0 min, 5 min, 10 min, and 15 min). The conditions for HBsAg-IC antibody dissociation were optimized based on an orthogonal design with five factors and four levels.

### Comparison of the PEG double-precipitation separation method with the traditional PEG precipitation separation method

Four free HBsAg-positive samples (2.68–182.92 IU/ml), 3 free anti-HBs-positive samples (23.89–897.32 mIU/ml of which 2 samples were from acute HBV-infected patients in recovery phase), 1 sample negative for both free HBsAg and free anti-HBs, and the prepared HBsAg-IC positive control I and positive control II were independently deposited and separated using the PEG double-precipitation separation method [19] and the traditional PEG precipitation separation method [22]. The CIC antibody dissociation technique developed by our group was then used to dissociate anti-HBs from HBsAg-ICs and the corresponding blank control. Finally, an Architect anti-HBs quantitative kit was used to detect anti-HBs, and the efficacy of precipitation and separation was compared between the two techniques.

### Methodological evaluation

#### Analytical sensitivity

A 0.9% NaCl solution was used for the serial twofold dilution of the prepared HBsAg-ICs (positive control I). Chemiluminescence (Abbott i2000 analyser) and an ELISA were employed to detect the anti-HBs in HBsAg-ICs after separation and dissociation from the HBsAg-ICs at different dilution titres, and the detectable anti-HBs concentration determined with the maximum dilution titre was used for the evaluation of detection sensitivity.

#### Reproducibility

The prepared HBsAg-ICs (positive control I) was diluted with 0.9% NaCl at ratios of 1:1 and 1:5. The original HBsAg-IC solution, the HBsAg-IC solution diluted to 1:2 and the HBsAg-IC solution diluted to 1:5 were then separated and dissociated 20 times each. Anti-HBs were measured to evaluate the precision of detecting anti-HBs after HBsAg-IC separation and dissociation.

#### Specificity

A total of 153 samples from the negative control group and 155 samples from the interference control group underwent HBsAg-IC separation and dissociation. Anti-HBs were detected to evaluate the specificity of the dissociation technique.

#### Dilution linearity

A 0.9% NaCl solution was used to dilute the prepared HBsAg-ICs (positive control I) to solutions corresponding to 100, 80, 60, 40, 20 and 0% of the original concentration, followed by HBsAg-IC separation and dissociation. Anti-HBs was then detected as a dependent variable, and the theoretical value served as an independent variable at each concentration. The linear regression equation and correlation coefficient (*r*) for anti-HBs after HBsAg-IC separation and dissociation were calculated.

#### Interference test

Seventy-five microlitres of the prepared HBsAg-ICs (positive control I) was independently added to 75 μl of HBV-M-negative serum (interference group) from patients (*n* = 155) with hypertriglyceridaemia, haemolysis, jaundice, IgG type hyperimmunoglobulinaemia, strong positivity for anti-HCV antibody, strong positivity for anti-HAV-IgM antibody, positivity for rheumatoid factor or antinuclear antibody, or strong positivity for CICs in the ELISA-C1q and 75 μl of 0.9% NaCl (*n* = 1). After mixing, HBsAg-ICs were separated and dissociated, and anti-HBs was then detected; 0.9% NaCl served as a reference. The extent of interference and the range of distribution were evaluated as follows:

1 $$ {\text{Extent}}\;{\text{of}}\;{\text{interfering}}\;(\% ) = \frac{{{\text{anti}} - {\text{HBs}}\;{\text{ in}}\;0.9\% \;{\text{NaCl}} - {\text{anti}} - {\text{HBs}}\;{\text{in}}\;{\text{interference}}}}{{{\text{anti}} - {\text{HBs}}\;{\text{ in }}0.9\% \, \;{\text{NaCl}}}} \times 100\% $$

#### Dissociation rate

The prepared HBsAg-ICs (positive control I) was separated and dissociated, and anti-HBs was measured 10 times. Anti-HBs at 473.6 mIU/ml determined after the preparation of the HBsAg-ICs served as a reference. The mean dissociation rate and the range of distribution of anti-HBs after HBsAg-IC separation and dissociation were calculated as follows:

2 $$ {\text{Dissociation}}\;{\text{ rate }}(\% ) = \frac{{{\text{ anti}} - {\text{HBs }}\;{\text{after}}\;{\text{ dissociation}} - {\text{anti}} - {\text{HBs}}\;{\text{ without}}\;{\text{ dissociation}}}}{{{\text{theoretical}}\;{\text{ anti}} - {\text{HBs}}\;{\text{ in}}\;{\text{ prepared}}\;{\text{ HBsAg}} - {\text{IC }}}} \times 100\% $$

### Recovery test

One hundred thirty-five microlitres mixed serum from healthy subjects (anti-HBs was < 10 IU/ml after HBsAg-IC separation and dissociation; *n* = 2) was mixed with 15 μl of prepared HBsAg-ICs (positive control I) or 15 μl of 0.9% NaCl. Anti-HBs was then measured 10 times after HBsAg-IC separation and dissociation. The mean recovery rate (%) and range were calculated as follows:

3 $$ {\text{Recovery}}\;{\text{rate }}(\% ) = \frac{{{\text{anti}} - {\text{HBs}}\;{\text{with}}\;{\text{adding}}\;{\text{control}} - {\text{anti}} - {\text{HBs}}\;{\text{with}}\;{\text{adding}}\;0.9\% \;{\text{NaCl}}}}{{{\text{theoretical}}\;{\text{anti}} - {\text{HBs}}\;{\text{in}}\;{\text{adding}}\;{\text{control }} \times {\text{ mean}}\;{\text{dissociation}}\;{\text{rate}}}} \times 100\% $$

### Stability

The CIC separating agents A and B, the CIC dissolving agent, the CIC antibody dissociation agent, and the CIC antibody dissociation neutralizing agent were independently incubated at 4 °C and 25 °C. The pH and osmolarity of these agents and the dissociation rate of the prepared HBsAg-ICs (positive control I) were measured at different time points (1, 3, 6, 9, 12, 15, 18 and 24 months). If the pH or osmolarity did not meet the standard, if the dissociation rate of anti-HBs from HBsAg-ICs was < 55% or if sediment was present in the agent, the reagent was disqualified.

### Dissociation conditions of ICs formed with different antigens

The above method and procedures were employed to investigate the dissociation conditions of HCV-ICs, HIV-ICs, INS-ICs and TG-ICs. The mean dissociation rate was measured to determine their dissociation conditions. The IC was determined according to the criteria given in Table 2.

### Preliminary application and comparison of the results of antigen detection in CICs with the CIC antigen dissociation technique

The CIC antigen dissociation technique was reported in a previous study [19]. The CIC antibody dissociation technique developed by our group was employed to separate and dissociate HBsAg-ICs (five HBV-M patterns) from 455 HBV-infected patients and 153 healthy volunteers, HCV-ICs from 126 patients with HCV infection, HIV-ICs from 32 patients with HIV infection, INS-ICs from 103 diabetes mellitus patients, and TG-ICs from 134 patients with hyperthyroidism, and the corresponding kits were employed to detect the antibodies after dissociation.

To verify the antibody detection results in the CICs of the above patients, we simultaneously determined the antigen in the CICs of the patients with the same disease using a previously described method [19]. The procedure steps were as follows: we precipitated and separated the CICs in the serum of patients with HCV, HIV, diabetes and hyperthyroidism; then, we dissociated the isolated CICs according to the reported methods in the literature [19] and determined the corresponding antigen in CICs. Finally, the detection rate (positive rate) of antigen in the CICs of patients with different diseases (HCV, HIV, diabetes and hyperthyroidism) was calculated. The reliability and effectiveness of the antibody dissociation technique for CICs were verified by comparing the consistency (Kappa value) between the antigen dissociation technique and the antibody dissociation technique.

### Statistical analysis

SPSS version 12.01 software was used for the statistical analysis. The linear regression equation and Pearson’s correlation between the measurement results and the theoretical values of anti-HBs after dissociation from HBsAg-ICs at each concentration were calculated. The consistency between the antibody dissociation technique and the antigen dissociation technique for CICs was evaluated by the Kappa value. A diagram of the conditions for antibody dissociation and a histogram of the positive rate of anti-HBs after dissociation from CICs were plotted using GraphPad Prism 5 software.

## Results

### Optimization of conditions for antibody dissociation from HBsAg-ICs

Tests with an orthogonal design experiment with five factors and four levels (Table 3) revealed that the optimal conditions for anti-HBs dissociation were a pH of 1.80, incubation in water for 5–10 min at 15 °C, shaking frequency > 60 cycles /min and detection within 10 min of neutralization. The HBsAg-IC antibody dissociation agent included 0.085 M NaCl, 0.02% ProClin 300 and 0.06 M glycine and HCl buffer system (pH 1.8). The HBsAg-IC antibody neutralization agent included 0.138 M NaCl, 0.07 M Tris solution, and 0.02% ProClin 300. The experimental results of the orthogonal design are shown in Table 3.

**Table 3:** The results of anti-HBs dissociation in HBsAg-IC with an orthogonal design

No.	pH	Temp (°C)	Dissociation time (min)	Oscillation frequency (times/min)	Place time (min)	Anti-HBs measure value (mIU/ml)
1	2.2	4	8	60	5	209.78
2	1.4	25	20	0	5	197.35
3	1.8	37	8	0	10	253.58
4	2.2	37	20	100	0	200.91
5	1.4	4	10	100	10	209.76
6	1.0	4	5	0	0	119.27
7	1.0	37	10	20	5	118.98
8	1.4	15	8	20	0	213.77
9	1.8	4	20	20	20	227.12
10	1.0	25	8	100	20	118.31
11	1.8	15	5	100	5	262.33
12	1.0	15	20	60	10	120.44
13	1.4	37	5	60	20	208.69
14	1.8	25	10	60	0	255.65
15	2.2	15	10	0	20	207.99
16	2.2	25	5	20	10	199.74
Level 1^a^	477.00	765.93	**790.03**	778.19	**789.60**	
Level 2^a^	829.57	**804.53**	**795.44**	759.61	**788.44**	
Level 3^a^	**998.68**	771.05	**792.38**	**794.56**	**783.52**	
Level 4^a^	818.42	782.16	745.82	**791.31**	762.11	

^a^The measured values at the different levels of each factor were calculated separately. The maximum sum value was the optimal level under this factor. Therefore, the optimal experimental conditions for anti-HBs dissociation in HBsAg-IC positive control II were as follows: CIC antibody dissociation agent with a pH of 1.80 (glycine-HCl buffer system), incubation for 5–10 min at 15 °C, oscillation frequency > 60/min and detection within 10 min after adding CIC antibody neutralizing agent (the highest statistical value with bold underline)

### Comparison of the PEG double-precipitation separation method with the traditional PEG precipitation separation method

For positive control I, positive control II and CIC-positive samples (samples 1, 3, 4, 5 and 6), the amounts of antigens and antibodies dissociated by the PEG double-precipitation separation method were significantly increased compared with those dissociated by the traditional method. When the sample contained a high level of free antigen or antibody (samples 3–6), the free antigen or antibody level of the PEG double-precipitation separation method was significantly lower than that of the traditional PEG precipitation method (*P* < 0.05). In the positive control I, II and CIC-positive samples (samples 1, 3–6), the amount of antibody dissociation was significantly increased over the traditional PEG precipitation method (*P* < 0.05). In addition, the PEG double-precipitation separation method increased the detection rate of antibodies in CICs (such as in sample 1 and sample 6) (*P* < 0.05). For CIC-negative samples (samples 2, 7 and 8), both methods exhibited similar dissociation capabilities (*P* > 0.05) (Table 4).

**Table 4:** The results of anti-HBs dissociation in HBsAg-IC by two PEG precipitation separation techniques (HBsAg: IU/ml, anti-HBs: mIU/ml)

Sample	Free HBsAg in serum	Free anti-HBs in serum	PEG double-precipitation separation technique					Traditional PEG precipitation separation technique				
			Measurement HBsAg	anti-HBs	Blank HBsAg	anti-HBs	Anti-HBs in CIC	Measurement HBsAg	anti-HBs	Blank HBsAg	anti-HBs	Anti-HBs in CIC
Positive control I	1.19	13.22	60.64	303.18	0.16	5.33	303.18	55.15	277.88	0.20	7.15	277.88
Positive control II	1.11	12.87	57.52	296.73	0.18	6.12	296.73	53.14	272.37	0.23	7.65	272.37
Sample 1	2.68	4.56	3.28	11.35	0.06	1.35	11.35	2.89	9.34	0.07	1.26	0.00
Sample 2	3.60	3.68	0.78	3.85	0.04	1.35	0.00	0.69	2.64	0.04	1.26	0.00
Sample 3	32.61	0.36	121.57	343.21	3.25	0.27	343.21	103.98	289.73	4.11	0.25	289.73
Sample 4	182.92	0.00	56.21	137.11	7.28	0.12	137.11	61.32	117.45	18.76	0.11	117.45
Sample 5	0.00	897.32	15.97	51.19	0.01	23.68	27.51	13.51	43.28	0.03	31.96	11.27
Sample 6	0.00	123.89	0.56	14.13	0.02	12.31	1.82	0.42	15.68	0.03	15.77	0.00
Sample 7	0.03	23.89	0.02	4.78	0.03	4.86	0.00	0.01	3.84	0.04	5.12	0.00
Sample 8	0.01	0.65	0.01	0.21	0.01 0.23	0.23	0.00	0.01	0.25	0.01	0.28	0.00

Control I, II: artificially prepared HBsAg-IC positive control I, II; theoretical level of anti-HBs: 473.6 mIU/ml; samples 1, 2, and 3: positive for serum free HBsAg, anti-HBe and anti-HBc; sample 4: positive for serum free HBsAg, HBeAg and anti-HBc; samples 5, 6, and 7: positive for serum free anti-HBs, anti-HBe and anti-HBc; samples 5 and 6: from acute HBV-infected patients in the recovery phase; and sample 8: negative for serum free HBsAg, anti-HBs, HBeAg, anti-HBe, and anti-HBc

### Methodological evaluation

The methodological evaluation of the measurement of anti-HBs in HBsAg-ICs is shown in Table 5.

**Table 5:** Methodological evaluation of anti-HBs dissociation in HBsAg-IC by the CIC antibody dissociation technique

Methodological parameters	Results	
Analytical sensitivity	Chemiluminescence	>10 mIU/ml
Reproducibility (CV)	High concentration (original)	4.38%
	Intermediate concentration (1:1)	5.31%
	Low concentration (1:5)	5.97%
Assay specificity	Interfere control (*n* = 155)	100%
	Negative control (*n* = 153)	100%
Dilution linearity	Regression equation	Y = 0.64*X* − 0.18, *Y*: actual anti-HBs in CICs; *X*: theoretical anti-HBs
	Correlation coefficient(*r*)	0.9932
Interference test	Extent of interfering	0.84% (− 1.25–2.17%)
Recovery	Recovery rate	95.4% (88.3–99.5%)
Dissociation rate	Dissociation rate	64.3%(56.1–68.0%)
Stability	Prepared HBsAg-IC (positive control)	4 °C for > 24 months
	CIC separating agent (A, B)	25 °C for > 24 months
	CIC dissolving agent	25 °C for > 24 months
	CIC antibody dissociation agent	4 °C for 12 months
	CIC antibody neutralizing agent	4 °C for > 24 months

### Dissociation conditions of CICs formed with different antigens

HCV-ICs, HIV-ICs, INS-ICs and TG-ICs were dissociated according to the procedures of HBsAg-IC dissociation. The results showed that the antibody dissociation conditions for CICs formed with different antigens were the same, but the dissociation rates of the antibody might have been slightly different. The conditions and mean dissociation rates are shown in Table 6.

**Table 6:** Antibody dissociation, determination conditions and dissociation rate in different immune complexes with the CIC antibody dissociation technique

CICs	Conditions for dissociation	Requirements for determination	Mean dissociation rate (%)
HCV-IC	pH 1.80, 15 °C, 5–10 min	Antibody measurement within 10 min after dissociation	–^a^
HIV-IC	pH 1.80, 15 °C, 5–10 min		–^a^
INS-IC	pH 1.80, 15 °C, 5–10 min		–^a^
TG-IC	pH 1.80, 15 °C, 5–10 min		42.1%

Qualitative test

### Preliminary application and comparison of the results of antigen detection in CICs with the CIC antigen dissociation technique

**Fig. 1:**
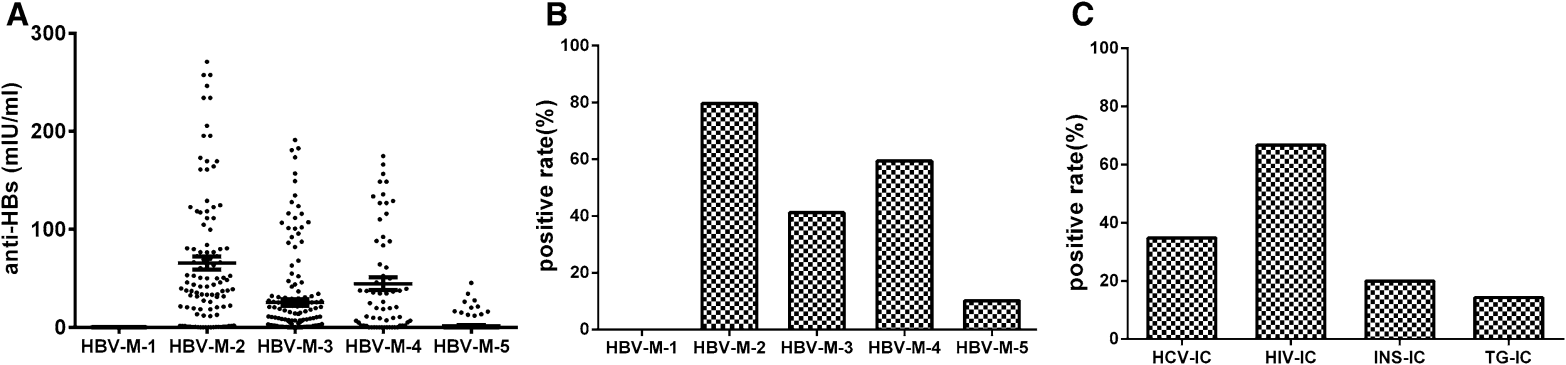
Scatter plot of antibody levels and histogram of antibody detection rates in HBsAg-IC, HCV-IC, HIV-IC, INS-IC, and TG-IC by the CIC antibody dissociation technique. **a** Scatter plot of anti-HBs levels in HBsAg-ICs with five HBV-M patterns by the CIC antibody dissociation technique. **b** Histogram of anti-HBs detection rates in HBsAg-ICs with five HBV-M patterns by the CIC antibody dissociation technique. **c** Histogram of antibody detection in HCV-ICs, HIV-ICs, INS-ICs and TG-ICs by the CIC antibody dissociation technique

The CIC antibody dissociation technique was used to separate and dissociate HBsAg-ICs with five HBV-M patterns in 455 HBV-infected patients and 153 healthy volunteers, HCV-ICs in 126 HCV-infected patients, HIV-ICs in 32 HIV-infected patients, INS-ICs in 103 diabetes mellitus patients, and TG-ICs in 134 patients with hyperthyroidism, and the corresponding antibodies were measured after dissociation. The histogram (Fig. 1a, b) shows that the detection rate of HBsAg-ICs (the positive rate of HBsAg-ICs) (anti-HBs, 79.63%) and the count of the HBsAg-ICs (anti-HBs, 65.68 ± 67.86 mIU/ml) in HBV-M-2 were the highest among the five HBV-M patterns. The detection rate of HBsAg-ICs (anti-HBs, 0%) and the count of the HBsAg-ICs (anti-HBs, 0.08 ± 0.04 mIU/ml) in HBV-M-1 were the lowest among the five HBV-M patterns. The histogram (Fig. 1c) shows that the positive rates for CICs formed with different antigens (HCV-IC, HIV-IC, INS-IC, and TG-IC) were different (14.3–66.7%). The corresponding antigens in HCV-ICs, HIV-ICs, INS-ICs and TG-ICs were simultaneously determined using the method described in the literature (the antigen dissociation technique for CICs) [19]. The consistency (Kappa value) in HCV-ICs, HIV-ICs, INS-ICs and TG-ICs between the antigen dissociation technique and the antibody dissociation technique for CICs was 0.947, 0.861, 0.912, 0.883(*P* < 0.05), respectively.

## Discussion

The CIC antibody dissociation technique is a pretreatment technique for the direct detection of antibodies after the precipitation, separation and dissociation of CICs. In this study, we developed and standardized a general and efficient CIC antibody dissociation technique that employed PEG double-precipitation separation and CIC dissociation and allowed the detection of antibodies in a variety of CICs.

Ultrafiltration, ultracentrifugation [23], immunomagnetic beads and PEG precipitation are the four main methods for the concentration and separation of CICs and viral granules [24, 25]. Among these methods, PEG precipitation is easy to perform in the lab. To exclude the interference of other confounding proteins in biosamples and to assure the sensitivity, specificity, repeatability, reliability and validity of CIC separation and dissociation, the antigens and antibodies in the CICs were separated and dissociated in a step-by-step process using multiple reagents. Compared with traditional PEG precipitation (Table 4), the appropriate amount of NaCl was added to increase the osmolarity (500–530 osm) in the double-precipitation separation method, which increased the efficiency of the IC separation and precipitation, decreased the coprecipitation of other confounding macromolecular proteins, and reduced the interference of free antigens and the adherence of free antibodies (Table 4). In the process of CIC dissociation and measurement, buffering and iso-osmolarity were employed to prevent the agents from damaging the antigens and antibodies and to ensure the repeatability, reliability and validity of the results, but these factors have not been seriously considered.

The CIC antibody dissociation technique may be used to assess both free-antibody-negative samples and free-antibody-positive samples that have a high concentration of free antibody. In addition, this technique may be used to concentrate and dissociate low levels of CICs due to the increased detection rate. In the present study, the dissociation rate was used to evaluate the dissociation technology, which is a new evaluation index. For HBsAg-ICs, the CIC antibody dissociation technique developed by our group achieved a dissociation rate as high as 64.3%. In addition, all the reproducibility, linearity, specificity, interference, recovery, stability and other methodological evaluation results met the requirements of clinical and scientific research (Table 5).

In our study, the polyclonal antibodies in the ICs formed by different antigens (HBsAg-ICs, HCV-ICs, HIV-ICs, INS-ICs and TG-ICs) were subjected to separation and dissociation and were then measured. The HBV-M1 was a healthy physical examination population without HBV infection. They had no HBV marker, and HBsAg-IC was detected in serum, so the results of anti-HBs in all HBsAg-IC were negative. This test shows that the CIC antibody dissociation technology had good specificity. HBV-M2, HBV-M3 and HBV-M4 were HBV infection patterns (free HBsAg positive), and HBV-M5 was the recovery period or a previous infection pattern of HBV (free HBsAg negative). The samples of these patterns were dissociated by the new technology, which detected anti-HBs at the highest level in HBV-M2 (Fig. 1a, b). These results agree with the literature [17]. As shown in Fig. 1c, the application of this technology in HCV-IC, HIV-IC, INS-IC and TG-IC also showed satisfactory results, but the rates of antibody detection in different disorders were quite different. We believe the difference in the detection rate (positive rate) of antibodies in CICs is mainly related to the pathological state of different diseases. These views have been confirmed in different stages of autoimmune glomerulonephritis [4], hepatitis B [8] and other diseases [5–7, 9–11]. Antibodies were not detected in some specimens of CIC, and there may be three explanations for their absence: (1) there was no CIC in the serum specimen of the patient; (2) the patients had CIC in the serum, but the content of CIC was low, and the precipitation separation technology and determination methods still do not meet that level of sensitivity; or (3) unknown reasons. These results indirectly reflect that the patients may have different clinical manifestations, pathological features and disease progression or chronic characteristics, which will be explored in a series of studies in the future.

Because of the separation, dissociation and determination of polyclonal antibodies in various immune complexes (HBsAg-IC, HCV-IC, HIV-IC, INS-IC, TG-IC, etc.), the dissociation conditions of these antibodies are essentially the same. Notably, the influence of monoclonal antibodies and the types of antibodies forming ICs (such as IgG, IgM and IgA) in the precipitation, separation and dissociation were not further investigated. More studies are required to elucidate these issues. In summary, a new CIC antibody dissociation technique was developed by our group and has the following advantages: (1) PEG double-precipitation separation is used to ensure a high efficiency of the precipitation and to effectively reduce the concentrations of interfering substances co-precipitating with the CICs (such as free antigens, free antibodies and other confounding macromolecular proteins); (2) during the dissociation, neutralization and measurement process, the CICs and dissociated antibodies are maintained in an isotonic environment, which minimizes damage to the antibodies and ensures the stability of the results; (3) the dissociation rate, which is more objective than specificity, is used to evaluate the performance of the dissociation technique; (4) the dissociation and subsequent measurement of antibodies are performed after the separation of the CICs, and this approach can be used in the assessment of both free-antibody-negative samples to increase the diagnostic sensitivity (increasing the detection rate of infectious diseases) and free-antibody-positive samples to prevent interference by free antibodies during the determination of antibodies in CICs after dissociation; (5) the technique can be used to concentrate and dissociate low levels of CICs, thus increasing the detection rate; and (6) the technique can be used as a routine method in clinical immunological examinations for monitoring disease progression and determining prognosis.

## Conclusion

We believe that this antibody dissociation technique for CICs can be widely applied to investigate the relationships between CICs and certain diseases associated with immune responses.

## Abbreviations

CICs: Circulating immune complexes
PEG: Polyethylene glycol
HBV: Hepatitis B virus
HBsAg: HBV surface antigen
anti-HBs: Antibody to hepatitis B surface antigen
HBeAg: Hepatitis Be antigen
anti-HBe: Antibody to hepatitis Be antigen
anti-HBc: Antibody to hepatitis B core antigen
HBsAg-ICs: Hepatitis B surface antigen-anti-HBs immune complexes (ICs)
HCV: Hepatitis C virus
HCV-ICs: HCV core-anti-HCV core antibody ICs
HIV: Human immunodeficiency virus
HIV-ICs: HIV P24-anti-HIV P24 antibody ICs
Ins: Insulin
Ins-ICs: Insulin-anti-insulin antibody ICs
TG: Thyroglobulin
TG-ICs: Thyroglobulin (TG)-anti-TG antibody ICs
ELISA: Enzyme-linked immunosorbent assay

## References

[1] Murphy K, Travers P, Walport M. Janeway’s Immunobiology. 8th ed. New York: Garland Science, Taylor & Francis Group.

[2] Iwasaki A, Medzhitov R (2015). Control of adaptive immunity by the innate immune system. Nat Immunol.

[3] Rojko JL, Evans MG, Price SA (2014). Formation, clearance, deposition, pathogenicity, and identification of biopharmaceutical-related immune complexes: review and case studies. Toxicol Pathol.

[4] Clynes R, Dumitru C, Ravetch JV (1998). Uncoupling of immune complex formation and kidney damage in autoimmune glomerulonephritis. Science.

[5] Stahl D, Sibrowski W (2005). Warm autoimmune hemolytic anemia is an IgM-IgG immune complex disease. J Autoimmun.

[6] Glazer E, Ejaz A, Coley CJ, Bednarek K, Theise ND (2007). Fibrin ring granuloma in chronic hepatitis C: virus-related vasculitis and/or immune complex disease?. Semin Liver Dis.

[7] Mustafa A, Nityanand S, Berglund L, Lithell H, Lefvert AK (2000). Circulating immune complexes in 50-year-old men as a strong and independent risk factor for myocardial infarction. Circulation.

[8] Du J, Tian P, Chen TY (2011). Progressive increase of serum circulating immune complexes and its significance in patients during the progression from chronic hepatitis B to hepatocellular carcinoma. Zhonghua Zhong Liu Za Zhi.

[9] Lopes-Virella MF, Baker NL, Hunt KJ (2011). Oxidized LDL immune complexes and coronary artery calcification in type 1 diabetes. Atherosclerosis.

[10] Li X, Zhang W, Yu HJ (2015). Clinical and pathological study on patients with primary antineutrophil cytoplasmic autoantibody-associated vasculitis with renal immune complex deposition. J Clin Rheumatol.

[11] Kang S, Keener AB, Jones SZ (2016). IgG-immune complexes promote B cell memory by inducing BAFF. J Immunol.

[12] Theofilopoulos AN, Dixon FJ (1980). Immune complexes in human diseases: a review. Am J Pathol.

[13] Wang S, Yang T, Zhang J, Xiao S, Peng X (2003). Analysis of Ig/Ig two-component-determined circulating immune complexes (TCIC) provide new insights into host immunity. Int Immunopharmacol.

[14] Nishanian P, Huskins KR, Stehn S, Detels R, Fahey JL (1990). A simple method for improved assay demonstrates that HIV p24 antigen is present as immune complexes in most sera from HIV-infected individuals. J Infect Dis.

[15] Aoyagi K, Ohue C, Iida K (1999). Development of a simple and highly sensitive enzyme immunoassay for hepatitis C virus core antigen. J Clin Microbiol.

[16] Zhang ZH, Li L, Zhao XP (2011). Elimination of hepatitis B virus surface antigen and appearance of neutralizing antibodies in chronically infected patients without viral clearance. J Viral Hepat.

[17] Takeda K, Maruki M, Yamagaito T (2013). Highly sensitive detection of hepatitis B virus surface antigen by use of a semiautomated immune complex transfer chemiluminescence enzyme immunoassay. J Clin Microbiol.

[18] Ohyama K, Ueki Y, Kawakami A (2011). Immune complexome analysis of serum and its application in screening for immune complex antigens in rheumatoid arthritis. Clin Chem.

[19] Dai Y, Hu Z, Chen Y (2018). A novel general and efficient technique for dissociating antigen in circulating immune complexes. Electrophoresis..

[20] de Carvalho CA, Partata AK, Hiramoto RM (2013). A simple immune complex dissociation ELISA for leishmaniasis: standardization of the assay in experimental models and preliminary results in canine and human samples. Acta Trop.

[21] Levinson SS, Goldman JO (1987). Evaluation of anti-C1q capture assay for detecting circulating immune complexes and comparison with polyethylene glycol-immunoglobulin G, C1q-binding, and Raji cell methods. J Clin Microbiol.

[22] Chia D, Barnett EV, Yamagata J (1979). Quantitation and characterization of soluble immune complexes precipitated from sera by polyethylene glycol (PEG). Clin Exp Immunol.

[23] Oda M, Uchiyama S, Noda M (2009). Effects of antibody affinity and antigen valence on molecular forms of immune complexes. Mol Immunol.

[24] Summa M, von Bonsdorff CH, Maunula L (2012). Evaluation of four virus recovery methods for detecting noroviruses on fresh lettuce, sliced ham, and frozen raspberries. J Virol Methods.

[25] Lee KB, Lee H, Ha SD, Cheon DS, Choi C (2012). Comparative analysis of viral concentration methods for detecting the HAV genome using real-time RT-PCR amplification. Food Environ Virol..

